# Patient-reported outcomes following primary total hip arthroplasty in Crowe type III or IV developmental dysplasia are comparable to those in Crowe type I: a case-control study of 96 hips with intermediate-term follow-up

**DOI:** 10.1186/s12891-020-03371-6

**Published:** 2020-06-03

**Authors:** Ken Ueoka, Tamon Kabata, Yoshitomo Kajino, Daisuke Inoue, Takaaki Ohmori, Takuro Ueno, Junya Yoshitani, Yuki Yamamuro, Hiroyuki Tsuchiya

**Affiliations:** Department of Orthopaedic Surgery, Graduate School of Medical Science, Kanazawa University, 13-1 Takaramachi, Kanazawa City, Ishikawa Prefecture 920-8641 Japan

**Keywords:** Total hip arthroplasty, Crowe classification, Satisfaction, Patient reported outcome, Case control study

## Abstract

**Background:**

A few previous studies have investigated patient satisfaction after total hip arthroplasty (THA) according to the degree of pelvic deformity. This study compared patient-reported outcomes after primary THA for Crowe types III, IV and I dysplasia.

**Methods:**

This retrospective, single-center, single-surgeon case-control study included patients who underwent primary THA between 2008 and 2016. We sent postal questionnaires to 38 patients with Crowe type III and IV dysplasia. Among the questionnaire respondents, 23 patients, excluding those with a follow-up period of < 1 year, were enrolled as the H group. The control group included 46 patients with Crowe type I, matched for sex, age, body mass index and surgical approach. To investigate the influence of femoral shortening osteotomy, the H group was divided according to whether femoral shortening osteotomy was performed. Ten patients underwent THA with femoral shortening osteotomy (FO group), while 12 patients underwent THA without femoral shortening osteotomy (N-FO group). Patient demographics, mean follow-up period, surgical information, pre- and postoperative leg length discrepancy (LLD), and perioperative complications were investigated. Clinical evaluations were performed using the Japanese Orthopaedic Association (JOA) scores, 36-item short-form survey (SF-36), net promotor score (NPS), visual analogue scale (VAS), and questionnaires. The VAS and SF-36 scores were determined only at final follow-up.

**Results:**

The H and control groups were not significantly different in the postoperative JOA scores and SF-36. In the H group, VAS at the final follow-up was significantly higher, and significantly more patients felt that postoperative rehabilitation was serious, expressing that they underwent THA for LLD correction. In addition, the VAS scores in the FO group was higher than those in the N-FO group. Postoperative LLD was significantly greater in the H group than in the control group. Each group had an NPS of > 50.

**Conclusion:**

The postoperative VAS score was higher in Crowe type III and IV dysplasia than in Crowe type I dysplasia, but no significant differences were detected in the postoperative satisfaction, JOA score, and SF-36 score. These findings may help explain the effects of THA preoperatively to patients with Crowe type III and IV dysplasia.

**Level of evidence:**

Therapeutic Level 3b.

## Background

The long-term outcomes of total hip arthroplasty (THA) have been excellent [4, 27]. THA is known to be associated with good satisfaction in terms of patient-reported outcome measures (PROMs) [9, 16]. THA for high hip dislocation was thought to be beyond surgical correction in the 1970s [2]. In recent years, with the introduction of femoral shortening osteotomy, some papers have reported relatively stable outcomes [23, 33, 36].

In general, there are various indications for THA, ranging from relatively mild to severe pelvic deformity, such as high hip dislocation. Patients with mild deformity experience pain and have restricted range of motion (ROM), which is likely to be the chief complaint [1]. On the other hand, patients with severe deformity may suffer from not only pain but also leg length discrepancy and joint contractures, as well as consequent changes in posture.

Therefore, we hypothesized that the patient-reported outcomes, expectations, and dissatisfaction for THA may vary when the chief complaint is different, depending on the degree of pelvic deformity. If the preoperative expectation is not met, satisfaction declines [7, 12]. It is important to know patients’ expectations before surgery. In addition, investigating and improving the points of dissatisfaction after surgery may lead to increased satisfaction.

The purpose of this study was to investigate clinical outcomes, including PROMs after THA for high hip dislocation, of Crowe classification type III and IV dysplasia [5] in comparison with those of Crowe type I dysplasia.

## Methods

### Patients and study design

This retrospective case-control study assessed patients who underwent primary THA at a single institution between 2008 and 2016. During the study period, our institution performed 661 primary THAs. All data for this study were obtained from the hospital archive system.

**Fig. 1:**
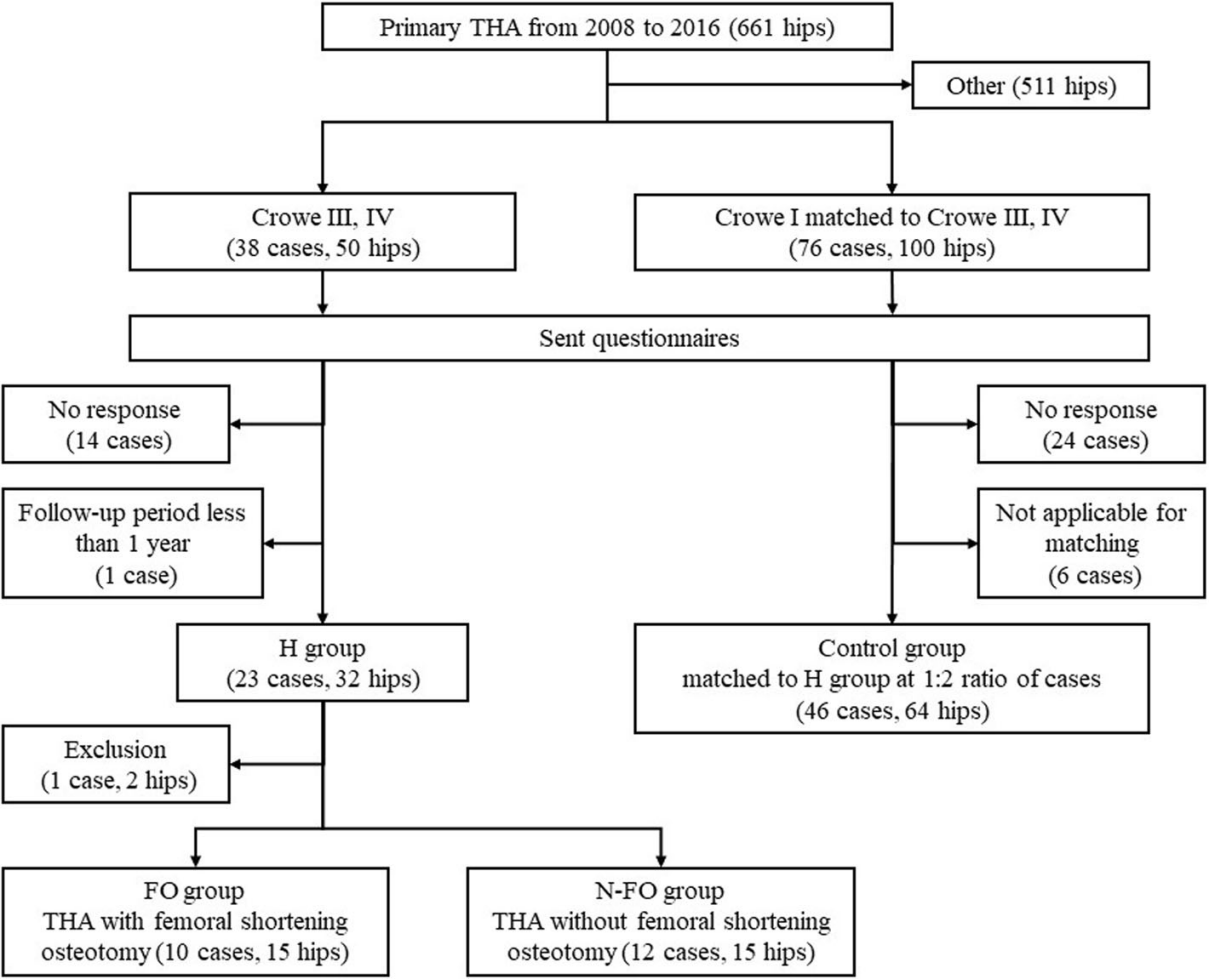
Flow chart of the study design. Twenty-three patients (32 hips) who underwent THA for Crowe type III/IV were enrolled as the high hip dislocation group (H group). The control group was formed by recruiting data-matched controls per patient in the H group. Data-matching involved matching for age (±10 years), sex, body mass index (±5 kg/m^2^), and surgical approach (posterior approach). The patients in the H group were divided according to whether femoral shortening osteotomy was performed (FO group) or not (N-FO group). THA: total hip arthroplasty; H group: high hip dislocation group

We sent postal questionnaires to 38 patients (50 hips) who underwent primary THA for high hip dislocation (Crowe type III and IV dysplasia) to evaluate postoperative satisfaction. Among the questionnaire respondents, 23 patients (32 hips), excluding those with a follow-up period of < 1 year after THA, were enrolled as part of the high hip dislocation group (H group). For the control group, we included 46 patients (64 hips) who underwent primary THA for Crowe type I dysplasia (Fig. 1). The control group was formed by recruiting data-matched controls per patient in the H group. Data matching involved matching for age (±10 years), sex, body mass index (±5 kg/m^2^), and surgical approach (posterior approach). To investigate the influence of femoral shortening osteotomy, the H group was divided according to whether femoral shortening osteotomy was performed or not. Ten patients (15 hips) underwent THA with femoral shortening osteotomy (FO group), while 12 patients (15 hips) underwent THA without femoral shortening osteotomy (N-FO group). One patient (2 hips) who underwent THA with femoral shortening osteotomy on one side and without on the other side was excluded when considering the influence of femoral shortening osteotomy.

### Surgical information

**Fig. 2:**
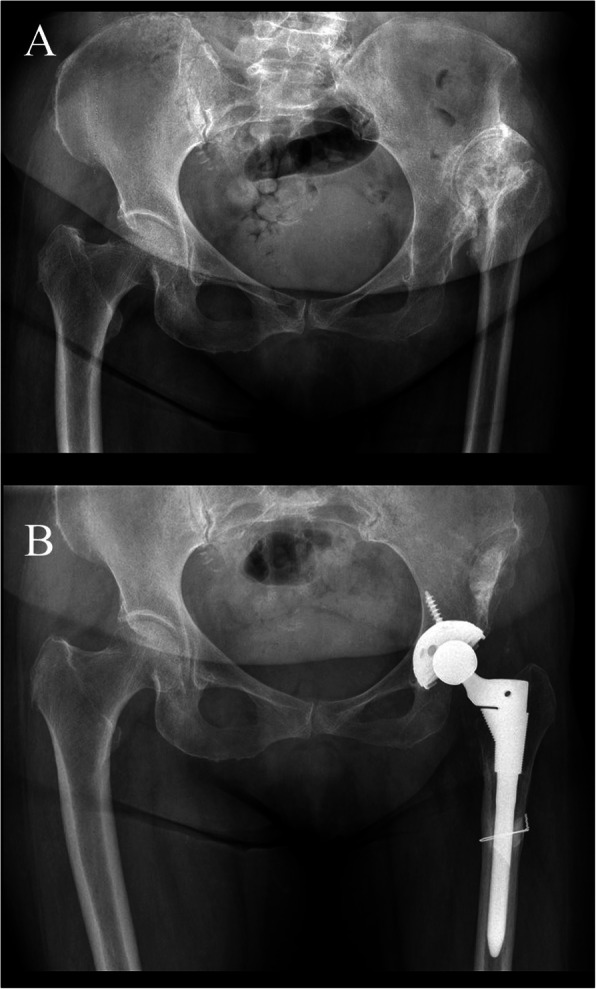
A 71-year-old woman with left high hip dislocation. Preoperative (**a**) and postoperative (**b**) radiographs at 5-year follow up

All operations were performed by a single senior surgeon using a posterior approach in a lateral decubitus position under general anesthesia. For cases with acute limb lengthening of > 40 mm at preoperative planning, THA with femoral shortening osteotomy (double chevron osteotomy) was performed (Fig. 2). Femoral shortening osteotomy was performed below the level of the lesser trochanter. The longitudinally split fragments from the resected femur were placed around the osteotomy site as a structural allograft. Morselized cancellous bone, which was obtained from the resected femoral head, was grafted to accelerate bone union at the osteotomy site.

Preoperative planning was performed for all THAs in both groups with the use of a computed tomography (CT)-based three-dimensional (3-D) templating and navigation software (CT-based Hip, version 1.0 or 1.1; Stryker Navigation, Freiburg, Germany). The cup was implanted with press-fit fixation with the assistance of the navigation system. The cup was basically implanted at the level of the true acetabulum. The main target of the cup orientation angle was at an anatomical inclination of 40° and anteversion of 20°. In both groups, all femoral components were implanted without the navigation system.

### Clinical evaluations

Clinical evaluations were performed using the patients’ demographics, Japanese Orthopaedic Association hip score (JOA score) [14], 36-item short-form health survey (SF-36), visual analogue scale (VAS), and the results of the unique questionnaire that was developed for the evaluation of patient-reported outcomes. The JOA score was evaluated prior to THA and at the time of final follow-up. The SF-36 and VAS scores were evaluated only at the time of the final follow-up and were enclosed in the questionnaires sent to the patients. The JOA score consists of four items: pain, ROM, gait, and activities of daily living, which are assessed by physicians. The total score is 100 points, with a higher score indicating higher hip function. In Japan, the JOA score is a common tool for clinical evaluation and is widely used [8]. There are reports that the JOA score and HHS are strongly correlated [15].

Leg length discrepancy (LLD) was measured from pre- and postoperative CT images (LightSpeed VCT; GE Medical Systems, Milwaukee, WI, USA) using the CT-based 3-D templating software (ZedHip; Lexi, Co., Ltd., Tokyo, Japan). In this study, LLD was defined as the difference in distance from the anterior superior iliac spine to the midpoint of the femoral condyle.

We obtained CT images 4 weeks prior to surgery and about 1 week after surgery.

CT images were acquired for 3-D templating preoperatively and for confirming the cup position postoperatively in other studies [34, 35].

### Questionnaire

**Fig. 3:**
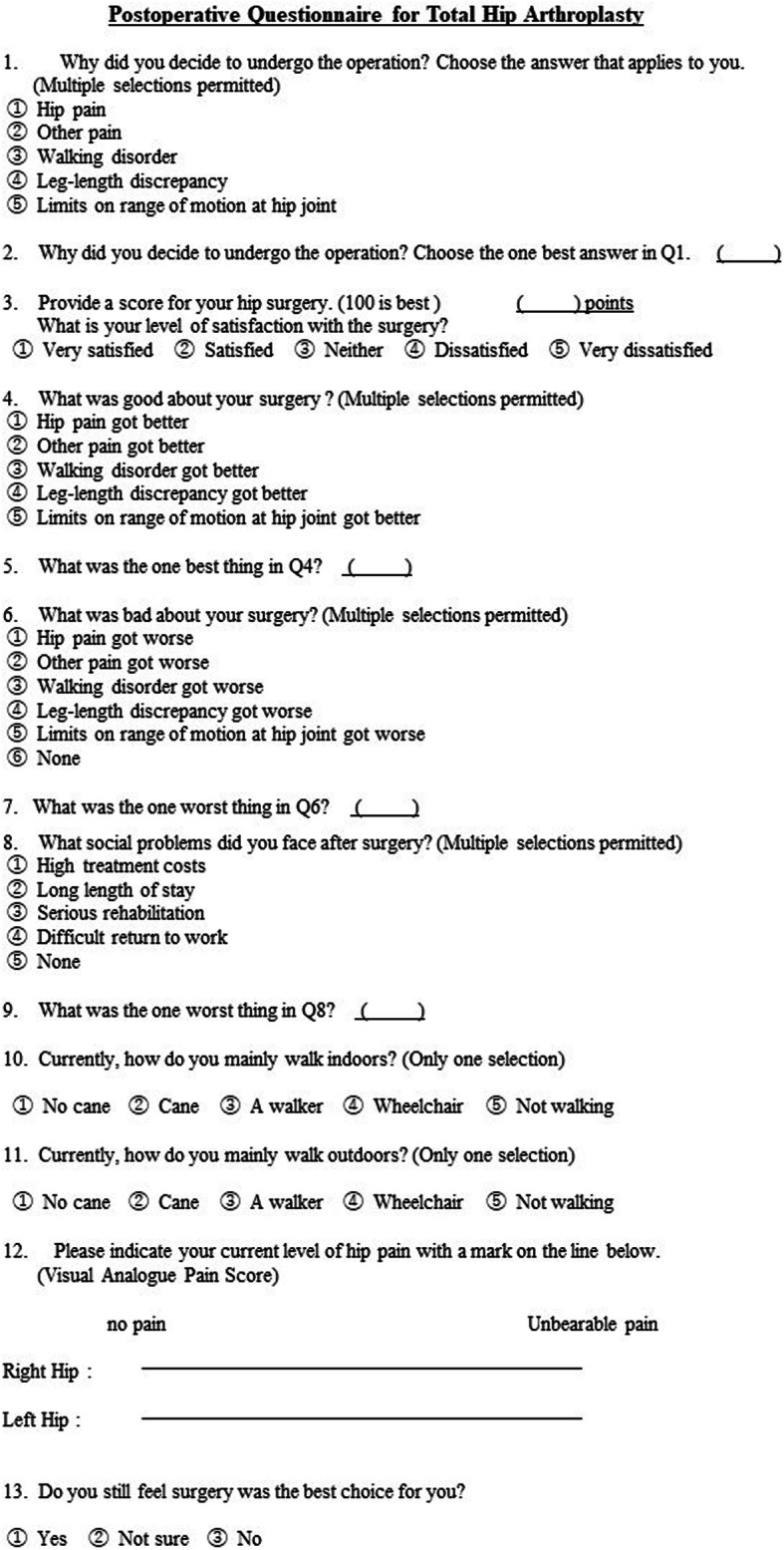
Sample postoperative questionnaire for THA patients. We sent postal questionnaires to all the patients enrolled in this study. The questionnaire consisted of 13 questions. The VAS was incorporated into the questionnaire. The SF-36 was enclosed in the questionnaire

The questionnaire consisted of 13 questions, which we developed for this study (Fig. 3). The contents included the reason for deciding to undergo the operation, the degree of satisfaction with the surgery (with a 0- to 100-point scale, for the patient to fill out themselves), positive or negative points about the surgery, social troubles, walking level, and VAS score. The last question was “Do you still feel that surgery was the best choice for you?” Missing data on the questionnaire were completed, where possible, via telephone interviews.

### Statistical analyses

Based on a previous report [30], we suggested that the minimal clinically important difference in JOA score was 10 points and the standard deviation (SD) was approximately 15 points. A power analysis suggested that 82 hips would be required to detect a clinically significant difference in the JOA score, with 80% power and 5% α error.

On the basis of the results of the 0- to 100-point scale, we further analyzed patient satisfaction using the net promoter score (NPS). The NPS is originally introduced across service industries to evaluate a consumer satisfaction [26]. It has also been used to assess patient satisfaction in orthopedic surgery [13, 32]. Form the original assessment of the NPS [26], on the 0- to 100-point scale, patients with scores > 90 were classified as “promoters”; those with scores between 70 and 89, were classified as “passives”; and those with scores < 70, as “detractors.” Then, the NPS was calculated by subtracting the percentage of “detractors” from the percentage of “promoters.” NPSs > 50 were considered good outcomes [26].

Statistical analyses were performed using a statistical software program (SPSS version 24.0 software for Windows; SPSS, Inc., Chicago, IL, USA). Group comparisons for quantitative data (e.g., patient demographics, SF-36 score, and VAS score) were performed using unpaired *t* tests, whereas categorical data (e.g., results of the questionnaire) were compared using the chi-square or Fisher exact test. A p-value of < 0.05 was considered statistically significant.

## Results

### Patient information

Patient information is summarized in Table 1. In the H group, 14 patients (19 hips) were classified with Crowe type IV dysplasia, while nine patients were classified with Crowe type III (13 hips). Among those with Crowe type IV, 7 hips had high hip dislocation in the gluteal muscle and 11 cases (16 hips) were treated with THA with femoral shortening osteotomy. Pre- and postoperative LLD ranged from 24.9 ± 13.9 mm to 9.4 ± 7.7 mm in the H group and from 8.1 ± 5.6 mm to 3.0 ± 2.1 mm in the control group (p < 0.001). Preoperative LLD in the FO group was significantly longer than that in the N-FO group, but no significant difference in postoperative LLD was detected. The postoperative LLD in one-sided THA with femoral shortening osteotomy was 18.7 ± 7.9 mm.

**Table 1:** Patient demographics

	H group (n = 32 hips)	Control (n = 64 hips)	***P***-value	FO group (n = 15 hips)	N-FO group (n = 15 hips)	P-value
Number of patients	23	46	–	10	12	–
Sex (male/female)	2/21	4/42	0.686	2/8	0/12	0.195
Unilateral/bilateral	14/9	28/18	1.000	5/5	9/3	0.221
Age (years)	65.3 ± 6.3	64.5 ± 8.4	0.864	69.3 ± 4.5	60.8 ± 6.2	**0.002**
Height (cm)	146.1 ± 6.5	157.3 ± 10.4	**<0.001**	142.8 ± 6.4	150.6 ± 5.4	**0.007**
Weight (kg)	47.9 ± 8.2	58.2 ± 12.4	**<0.001**	48.0 ± 12.0	48.8 ± 7.1	0.849
BMI (kg/m^2^)	22.0 ± 3.4	23.5 ± 3.2	0.202	23.5 ± 5.4	21.5 ± 2.8	0.284
Diagnosis (number of hips)						
Dysplasia	31	64	0.333	14	15	0.500
Other	1	0	0.333	1	0	0.500
Average follow-up period (years)	5.8 ± 2.6	5.4 ± 2.4	0.557	5.4 ± 2.8	6.3 ± 2.8	0.443
Intraoperative blood loss (mL)	465.9 ± 295.6	245.5 ± 143.0	**<0.001**	596.0 ± 363.9	355.0 ± 212.1	0.067
Surgery time (min)	252.4 ± 108.0	149.5 ± 28.3	**<0.001**	350.0 ± 124.4	177.7 ± 42.8	**0.002**
LLD-preoperative (mm)	24.9 ± 13.9	8.1 ± 5.6	**<0.001**	31.9 ± 16.0	18.4 ± 10.0	**0.026**
LLD-postoperative (mm)	9.4 ± 7.7	3.0 ± 2.1	**0.041**	12.5 ± 9.4	7.5 ± 5.6	0.140
Complications						
Infection	2	0	0.109	0	2	0.286
Dislocation	1	1	0.716	1	0	0.455
Intraoperative fracture	0	1	0.536	0	0	**–**
Preoperative JOA score (points)						
Pain	15.5 ± 7.0	16.1 ± 6.5	0.670	15.7 ± 7.9	16.0 ± 6.1	0.903
ROM	10.8 ± 4.7	12.4 ± 4.0	0.081	12.4 ± 5.2	9.4 ± 3.6	0.056
Gait	8.4 ± 2.9	9.3 ± 3.4	0.242	8.0 ± 2.5	9.9 ± 3.3	0.559
ADL	10.9 ± 3.9	11.3 ± 3.5	0.641	10.8 ± 4.3	11.1 ± 3.6	0.931
Total	45.8 ± 11.1	49.3 ± 10.6	0.139	47.1 ± 12.0	45.2 ± 10.4	0.579
Postoperative JOA score (points)						
Pain	37.2 ± 3.0	37.7 ± 3.3	0.439	37.23 ± 3.1	37.3 ± 3.1	1.000
ROM	17.4 ± 2.6	18.0 ± 2.6	0.309	17.1 ± 3.3	17.7 ± 1.8	0.596
Gait	16.6 ± 3.8	17.6 ± 3.0	0.191	15.8 ± 5.0	17.7 ± 2.2	0.330
ADL	16.3 ± 4.1	17.6 ± 2.7	0.096	15.5 ± 4.8	17.1 ± 3.4	0.314
Total	87.4 ± 9.1	90.8 ± 8.7	0.080	85.7 ± 10.5	89.2 ± 7.7	0.327
Postoperative SF-36 (points)						
PF	73.3 ± 19.7	70.1 ± 22.3	0.568	75.0 ± 14.7	71.7 ± 24.1	0.696
RP	81.3 ± 19.8	79.2 ± 24.2	0.728	82.9 ± 16.8	79.7 ± 22.9	0.705
BP	72.0 ± 22.5	63.6 ± 23.2	0.158	71.0 ± 21.5	72.9 ± 24.4	0.844
GH	59.2 ± 19.3	60.5 ± 16.7	0.780	59.2 ± 19.3	60.5 ± 16.7	0.624
VT	66.6 ± 19.0	65.9 ± 15.4	0.875	68.8 ± 18.3	64.6 ± 20.2	0.610
SF	88.6 ± 18.8	87.2 ± 17.0	0.763	89.8 ± 9.4	87.5 ± 25.0	0.780
RE	83.0 ± 21.2	83.0 ± 21.3	1.000	86.4 ± 20.2	79.9 ± 22.6	0.477
MH	72.2 ± 20.7	77.5 ± 15.1	0.228	71.4 ± 21.8	72.9 ± 20.6	0.862

The control group included 46 patients (64 hips) who underwent primary THA for Crowe I dysplasia, matched for age, sex, BMI, and surgical approach

Values are expressed as means ± SD or as numbers (n). P-values in bold indicate statistical significance (P < 0.05)

*H group* high hip dislocation group, *FO group* femoral shortening osteotomy group, *N-FO group* non-femoral shortening osteotomy group, *THA* total hip arthroplasty, *BMI* body mass index, *LLD* leg length discrepancy, *JOA* Japanese Orthopaedic Association, *ROM* range of motion, *ADL* activities of daily living, *SF-36* Short form-36, *PF* Physical Functioning, *RP* Role Physical, *BP* Bodily Pain, *GH* General Health, *VT* Vitality, *SF* Social Functioning, *RE* Role Emotional, *MH* Mental Health, *SD* standard deviation

### Complications

In the H group, infection at the site of the central venous catheter occurred in one patient. The infection was improved with intravenous antibiotic therapy without implant removal. Periprosthetic joint infection occurred in one patient, and two-stage revision THA was performed. Recurrence of infection was not observed. Temporary sciatic nerve paralysis occurred postoperatively in one patient, and facial nerve paralysis due to a lengthy surgery in the lateral decubitus position occurred in one patient. The patient recovered within 3 months without any functional defects. Postoperative dislocations occurred in one patient, around 2 weeks after surgery.

In the control group, intraoperative greater trochanter chip fractures were identified in two patients, which were treated using cerclage wires and healed without further sequelae. Postoperative dislocations occurred in one patient, around 5 years after surgery.

We also did not recognize obvious septic or aseptic loosening of implants that required revision THA in the radiographic evaluation at the final follow-up. In all the patients in whom femoral shortening osteotomy was performed, the osteotomy site healed without any complications by the time of final follow-up.

### PROMs

In the H group, the JOA score was 45.8 ± 11.1 points preoperatively, which significantly improved to 87.4 ± 9.1 points at the final follow-up (p < 0.001). In the control group, the scores were 49.3 ± 10.6 and 90.8 ± 8.7, pre- and postoperatively, respectively (p < 0.001). The percentage of patients with improvement in postoperative JOA score of ≥30 points was 84.4% (27/32 hips) in the H group and 82.8% (53/64 hips) in the control group. In both groups, no significant differences were found in the preoperative and postoperative JOA scores, including the subscale scores (Table 1).

The SF-36 scores, including the subscale scores, at final follow-up showed no significant difference in either group (Table 1). No significant differences in JOA and SF-36 scores were detected between the FO and N-FO groups. The NPS is shown in Table 2. Each group had the NPS of > 50.

**Table 2:** The Net promoter score in each group

Groups	Promoters	Passives	Detractors	NPS
H group (n = 23)	69.5% (n = 16)	26.1% (n = 6)	4.3% (n = 1)	**65**
Control (n = 46)	80.4% (n = 37)	10.9% (n = 5)	8.7% (n = 4)	**72**
FO group (n = 10)	70.0% (n = 7)	20.0% (n = 2)	10.0% (n = 1)	**60**
N-FO group (n = 12)	75.0% (n = 9)	25.0% (n = 3)	0% (n = 0)	**75**

On the 0–100 scale, patients scoring above 90 were classified as “promoters”, between 70 and 89 were classified as “passives” and under 70 were classified as “detractors”. The NPS was calculated by subtracting the percentage of “detractors” from the percentage of “promoters”

*NPS* Net promoter score, *FO group* femoral shortening osteotomy group, *N-FO group* non-femoral shortening osteotomy group

### Questionnaire

Satisfaction with THA was 90.3 ± 11.3 points in the H group and 91.5 ± 12.5 points in the control group. The satisfaction rate was approximately 95.6% (22/23 cases) in the H group and 93.5% (43/46) in the control group. No significant difference was found between the groups in terms of satisfaction (Table 3). Similarly, no significant difference was detected between the FO and N-FO groups. However, satisfaction in the FO group tended to be low (p = 0.057; Table 3).

**Table 3:** Questionnaire results

	H group (23 cases)	Control (46 cases)	P-value	FO group (10 cases)	N-FO group (12 cases)	P-value
Q1. The reason for receiving THA						
1. Hip pain	19	41	0.468	7	11	0.293
2. Other pain	9	14	0.470	6	3	0.192
3. Walking disorder	18	40	0.487	8	9	1.000
4. LLD	13	9	**0.002**	5	8	0.666
5. Limits on ROM at hip joint	16	27	0.380	7	8	1.000
Q2. Primary complaint						
1. Hip pain	15	32	0.715	6	8	0.546
2. Other pain	0	3	0.546	0	0	–
3. Walking disorder	6	5	0.161	4	2	0.348
4. LLD	1	0	0.333	0	1	0.545
5. Limits on ROM at hip joint	1	6	0.411	0	1	0.545
Q3. Satisfaction following THA						
Points	90.3 ± 11.3	91.5 ± 12.5	0.680	84.8 ± 13.2	94.0 ± 6.6	0.057
Rates, % (n/N)	95.6 (22/23)	93.5 (43/46)	0.593	90.0 (9/10)	100.0 (12/12)	0.455
Q4. Benefits of THA						
1. Hip pain subsided	19	43	0.211	7	11	0.226
2. Other pain subsided	6	12	0.071	3	3	0.583
3. Walking disorder improved	16	38	0.216	6	9	0.652
4. LLD improved	12	9	**0.006**	5	7	0.515
5. ROM improved	12	32	0.157	4	7	0.392
Q5. Best outcome						
1. Hip pain subsided	13	29	0.601	6	7	0.639
2. Other pain subsided	1	3	0.892	0	1	0.545
3. Walking disorder improved	6	8	0.527	2	3	0.594
4. LLD improved	2	0	0.108	1	1	0.714
5. ROM improved	1	6	0.411	1	0	0.455
Q6. Adverse outcomes						
1. Hip pain worsened	0	1	0.667	0	0	–
2. Other pain worsened	3	4	0.435	1	2	0.571
3. Walking disorder worsened	3	4	0.435	2	0	0.195
4. LLD worsened	0	1	0.667	0	0	–
5. Limits on ROM worsened	0	3	0.290	0	0	–
6. None	17	33	0.544	7	10	0.229
Q7. Worst outcome						
1. Hip pain worsened	0	1	0.667	0	0	–
2. Other pain worsened	3	4	0.435	2	1	0.429
3. Walking disorder worsened	3	4	0.435	2	0	0.195
4. LLD worsened	0	1	0.667	0	0	–
5. Limits on ROM worsened	0	3	0.290	0	0	–
6. None	17	33	0.544	6	11	0.105
Q8. Social problems after THA						
1. High treatment cost	2	2	0.596	0	2	0.286
2. Long length of stay	4	2	0.09	2	2	0.632
3. Serious rehabilitation	10	8	**0.02**	7	2	**0.017**
4. Difficult return to work	0	4	0.293	0	0	**–**
5. None	13	32	0.284	3	8	0.099
Q9. Worst social problem after THA						
1. High treatment cost	0	1	0.667	0	0	**–**
2. Long length of stay	2	2	0.596	0	2	0.286
3. Serious rehabilitation	10	7	**0.01**	7	2	**0.017**
4. Difficult return to work	0	4	0.293	0	0	**–**
5. None	11	32	0.079	3	8	0.099
Q10. Walking indoors						
1. No cane	20	44	0.202	7	12	0.078
2. Cane	3	2	0.202	3	0	0.078
3. A walker	0	0	–	0	0	–
4. Wheelchair	0	0	–	0	0	–
5. Not walking	0	0	–	0	0	–
Q11. Walking outdoors						
1. No cane	18	38	0.465	6	11	0.105
2. Cane	4	8	0.640	3	1	0.226
3. A walker	1	0	0.333	1	0	0.455
4. Wheelchair	0	0	–	0	0	**–**
5. Not walking	0	0	–	0	0	**–**
Q12. VAS score (mm)	14.2 ± 12.9	9.3 ± 7.5	**0.036**	23.1 ± 14.4	6.2 ± 8.0	**0.001**
Q13. THA was still the best choice?						
1. Yes	20	39	0.559	8	11	0.571
2. Not sure	3	5	0.538	2	1	0.571
3. No	0	2	0.441	0	0	–

Values are expressed as means ± SD, numbers (n), or percentages (n/N). P-values in bold indicate statistical significance (P < 0.05)

*H group* high hip dislocation group, *FO group* femoral shortening osteotomy group, *N-FO group* non-femoral shortening osteotomy group, *THA* total hip arthroplasty, *LLD* leg length discrepancy, *ROM* range of motion, *VAS* visual analogue scale

The VAS scores were significantly higher in the H group (14.2 ± 12.9 mm) than in the control group (9.3 ± 7.5 mm; p = 0.036; Table 3). In addition, the VAS scores in the FO group were higher than those in the N-FO group (Table 3).

Hip pain was the primary reason for undergoing THA, with 65.2% (15/23 cases) in the H group and 69.6% (32/46 cases) in the control group. However, when multiple selections were possible, 52% (12/23) and 20% (9/46) of the patients in the H and control groups underwent THA for LLD correction (p = 0.006; Table 3).

With regard to the most socially troubling aspect of receiving THA, 44% (10/23 cases) of the patients in the H group selected “serious rehabilitation,” which was higher than the corresponding number in the control group (17%, 8/46 cases). Of the 10 patients who selected “serious rehabilitation,” seven were in the FO group, which is a significantly higher number than that in the N-FO group. No significant difference was observed in walking ability between the groups. Around 80% of patients in both groups could walk alone outdoors (Table 3).

## Discussion

This study with a mean follow-up period > 5 years showed that postoperative satisfaction, JOA score, and SF-36 score of primary THA for Crowe type III and IV dysplasia were comparable with those for Crowe type I. The height of the H group was significantly shorter than that of the control group owing to the effect of high hip dislocation. In the H and FO groups, the intraoperative blood loss and surgery time were increased compared with the control and N-FO groups. No significant difference in perioperative complications was detected, but the results may change as the number of cases increases.

### PROMs

The postoperative JOA score was favorable without any significant difference between the H and control groups, and between the FO and N-FO groups. The JOA score in the patients with high hip dislocation was not much different from those in previous reports [17]. Although no significant difference was detected, the JOA score tended to be poor in the cases with femoral osteotomy (FO group).

The SF-36 scores, including the subscale scores, at the final follow-up showed no significant difference between the H and control groups, and between the FO and N-FO groups. Each subscale score was as good as, or even better than, those in previous reports [21, 10]. As will be described in the Limitations section, the reasons for this result were considered the possible selection bias in this study. In other words, in this study, only the patients who responded to the postal questionnaire were enrolled, and it is possible that cases with good postoperative outcomes were selectively analyzed. However, no significant difference was detected in postoperative SF-36 between the H and control groups, which suggests no significant difference in postoperative PROMs depending on the degree of preoperative pelvic deformity.

In addition, each group had a NPS of > 50 and was considered a good outcome. Hamilton et al. reported a NPS of 60 for joint replacement and individual scores of 71 and 49 for total hip replacement (THR) and total knee replacement (TKR), respectively [13]. From the above-mentioned results, we considered that the patients in the FO group with the lowest NPS (60) in this study were also satisfied with THA.

### Questionnaire

Questionnaires were provided to measure the patients’ postoperative satisfactions. The patients were allowed to choose one of the following five options: very satisfied, satisfied, neither, dissatisfied, and very dissatisfied. Then, the patients who selected “very satisfied” or “satisfied” were considered satisfied. This method of rating patient satisfaction through the selection of one of the five options is called the 5-point Likert scale, which has good measurement properties, validity, and reliability [6]. In addition, it is simple and available in many languages [28]. In this study, patient satisfaction was measured with two methods, the NPS and Likert scale. Therefore, we believe that the results on patient satisfaction were of high reliability.

In the H group, the VAS scores at the final follow-up were significantly higher than those in the control group, and more patients felt that postoperative rehabilitation was serious. When the H group was divided into the FO and N-FO groups, the VAS scores and number of patients who felt “serious rehabilitation” in the FO group were significantly higher. These results suggest that the FO group might have an adverse effect on the clinical outcomes in the H group. All the patients in the FO group had Crowe type IV dysplasia and had severe cases with acute limb lengthening of > 40 mm. Some reports indicated that the intensity of early postoperative pain increases the risk of chronic postsurgical pain [11, 25]. THA with femoral shortening osteotomy is an effective and reliable technique [20, 33, 36]. However, on the basis of this study, for patients who will undergo THA with femoral shortening osteotomy, it is desirable to explain before surgery that the postoperative rehabilitation will be more serious and pain may persist unlike in with normal cases.

Patients sometimes had low back pain before THA [3, 31], which was improved after THA [24, 37]. In this study, low back pain improved after surgery in all the cases. It was found that THA easily improved preoperative back pain even in patients with high hip dislocation. The chief complaints of patients with high hip dislocation in the gluteal muscle are often low back and buttock pains. Therefore, low back pain caused by malalignment due to pelvic deformity or LLD should be considered an important factor in determining the surgical indication for THA for Crowe type III and IV dysplasia.

### Leg length discrepancy

Many past reports indicated that a postoperative LLD of > 10 mm decreases the postoperative function and satisfaction of THA [18, 22]. In the H group, the patients strongly wanted to undergo LLD correction, probably because of the large difference in preoperative LLD. However, when one-sided THA with femoral shortening osteotomy for high hip dislocation was performed, the amount of LLD correction was limited, and the postoperative LLD was often > 10 mm. In this study, the postoperative LLD of one-sided THA with femoral shortening osteotomy was 18.7 ± 7.9 mm. Although a significant difference was not detected, it may have led to declined satisfaction in the FO group.

### Limitations

This study had several limitations. First, the number of cases was small owing to the rarity of high hip dislocation. Only a few cases were available for the investigation of the effects of femoral shortening osteotomy. If the number of hips is increased, the results of the questionnaire survey could show a significant difference. However, considering that high hip dislocation was extremely rare and that the study was performed at a single institution, this study had an adequate number of hips for analysis. Second, selection bias was possible because only the patients who answered the questionnaire were enrolled in this study. This fact could artificially inflate the proportion of satisfied or unsatisfied patients. The response rates in this study were not high enough at 61% (23/38 cases); however, this proportion was similar to those reported in previous survey studies [19, 29]. Third, the validity of the questionnaire used in this study had not been determined, and the presence of concurrent knee conditions at the time of the evaluation was overlooked. However, the questionnaire included the NPS, Likert scale, and VAS, which have been evaluated for their effectiveness in past reports [13, 28], and we considered that the questionnaire had a certain validity and reliability. Finally, preoperative PROMs, especially SF-36, had not been acquired, so the degree of improvement in surgery could not be investigated. However, the PROMs at the final follow-up were comparable between the H and control groups. Future studies that consist of more cases of high hip dislocation with a longer follow-up period are warranted to confirm the results of this study.

## Conclusions

This study revealed that postoperative satisfaction (include NPS), JOA score, SF-36 score, and walking ability after primary THA were comparable between Crowe type III/IV dysplasia and Crowe type I. Patients with Crowe type III and IV dysplasia strongly wanted to undergo LLD correction, and preoperative low back pain was as easy to improve after THA as in Crowe type I. However, the patients who underwent THA with femoral shortening osteotomy had higher VAS scores at the final follow-up, and more of these patients felt that postoperative rehabilitation was serious than the patients without femoral shortening osteotomy for high hip dislocation. These findings of this study may help explain the effects of THA preoperatively to patients with Crowe type III and IV dysplasia.

## Data Availability

All the data used and/or analyzed during this study are available upon reasonable request from the corresponding author.

## Abbreviations

CT: Computed tomography
JOA: The Japanese Orthopaedic Association
LLD: Leg length discrepancy
NPS: Net promoter score
PROMS: Patient-reported outcome measures
ROM: Range of motion
SD: Standard deviation
SF-36: 36-item short-form health survey
THA: Total hip arthroplasty
VAS: Visual analogue scale
